# Silencing of Ago-2 Interacting Protein SERBP1 Relieves KCC2 Repression by miR-92 in Neurons

**DOI:** 10.3390/cells11061052

**Published:** 2022-03-20

**Authors:** Christian Barbato, Paola Frisone, Laura Braccini, Simona D’Aguanno, Luisa Pieroni, Maria Teresa Ciotti, Caterina Catalanotto, Carlo Cogoni, Francesca Ruberti

**Affiliations:** 1 Institute of Biochemistry and Cell Biology, National Research Council CNR, Department of Sense Organs, University of Rome Sapienza, 00161 Roma, Italy; 2 Institute of Biochemistry and Cell Biology CNR, Campus A. Buzzati-Traverso, 00015 Monterotondo (RM), Italy; p.frisone@gmail.com (P.F.); mariateresa.ciotti@cnr.it (M.T.C.); 3 Department of Molecular Medicine, University of Rome Sapienza, 00161 Roma, Italy; braccini.laura@hotmail.it (L.B.); caterina.catalanotto@uniroma1.it (C.C.); 4 Preclinical Models and New Therapeutic Agents Unit, IRCCS Regina Elena National Cancer Institute, 00144 Rome, Italy; simona.daguanno@ifo.it; 5 Department of Experimental Neuroscience, Proteomics and Metabolomics Unit, IRCCS-Fondazione Santa Lucia, 00143 Rome, Italy; l.pieroni@hsantalucia.it

**Keywords:** microRNA, RNA-binding protein, Ago2, KCC2, SERBP1, RNA-induced silencing complex (RISC)

## Abstract

RNA-binding proteins (RBPs) play important roles in modulating miRNA-mediated mRNA target repression. Argonaute2 (Ago2) is an essential component of the RNA-induced silencing complex (RISC) that plays a central role in silencing mechanisms via small non-coding RNA molecules known as siRNAs and miRNAs. Small RNAs loaded into Argonaute proteins catalyze endoribonucleolytic cleavage of target RNAs or recruit factors responsible for translational silencing and mRNA target destabilization. In previous studies we have shown that KCC2, a neuronal Cl (−) extruding K (+) Cl (−) co-transporter 2, is regulated by miR-92 in neuronal cells. Searching for Ago2 partners by immunoprecipitation and LC-MS/MS analysis, we isolated among other proteins the Serpine mRNA binding protein 1 (SERBP1) from SH-SY5Y neuroblastoma cells. Exploring the role of SERBP1 in miRNA-mediated gene silencing in SH-SY5Y cells and primary hippocampal neurons, we demonstrated that SERBP1 silencing regulates KCC2 expression through the 3′ untranslated region (UTR). In addition, we found that SERBP1 as well as Ago2/miR-92 complex bind to KCC2 3′UTR. Finally, we demonstrated the attenuation of miR-92-mediated repression of KCC2 3′UTR by SERBP1 silencing. These findings advance our knowledge regarding the miR-92-mediated modulation of KCC2 translation in neuronal cells and highlight SERBP1 as a key component of this gene regulation.

## 1. Introduction

RNA-mediated gene silencing is a mechanism modulating gene expression at post-transcriptional level, which acts via small double-strand RNA molecules known as short interfering RNAs (siRNAs) and microRNAs (miRNAs) [1]. Argonaute family proteins are a fundamental component of effector complexes (RISC/miRNP complex) of silencing mechanisms by siRNAs and miRNAs. The RNA-induced silencing complex (RISC), minimally composed of Ago and miRNAs, drives mRNA target repression by association of the miRNA strand to partially complementary sites usually present within the 3′ untranslated region (UTR) of the target [2]. An increasing amount of evidence in different organisms indicates that Argonaute proteins interact with a plethora of protein-binding partners, leading to miRNA-mediated silencing pathways [3]. Most studies on the role of RNA-mediated gene silencing in the nervous system belong to the exploration of single miRNAs or families of miRNAs involved in the regulation of gene expression in neuronal subtype cells, in selected cellular compartments, such as nucleus or cytoplasm, dendrites or axons and at specific stages of brain development. In these studies, the principal role of Ago2 bound to miRNAs and its ability to compose the ‘minimal RISC’ was evaluated [4]. Nevertheless, Ago2 interacting proteins have been found to modulate RISC function in neuronal cells. A few examples are as follows: Nova1 binds Ago2 and positively regulates neuronal miRISC activity [5]; Pick1 interacts with Ago2 and drives its localization at endosomal compartments in dendrites of hippocampal neurons and may inhibit miRISC function [6]; NMDA-mediated increase of the interaction between Ago2 and GW182, via Akt-mediated phosphorylation of Ago2 at S387, was implicated in a stronger miRNA-mediated translational repression of a particular mRNA target [7]. Due to the relevance of Ago2 protein complex composition, we aimed to find partners of the protein complexes containing human Ago2 (hAgo2) in SH-SY5Y neuroblastoma cells [8].

SERBP1 (Serpine mRNA Binding Protein 1) was initially identified as a protein that binds to the 3′ UTR of the mRNA encoding plasminogen activator inhibitor type I (PAI-1 or Serpine1) and regulates mRNA stability [9]. Besides its main role in post-transcriptional regulation, SERBP1 has been shown to be involved in many cellular processes including DNA cleavage and recombination [10,11], and chromatin remodeling and transcription [12,13]. Among RNA-binding proteins, SERBP1 shows a specific capacity to work inside and outside the nucleus [14,15]. The translational regulation by SERBP1 may also be mediated through SPINDLIN1, which constitutes a ribonucleoprotein complex with SERBP1 via its Tudor-like domain [16]. Recently, SERBP1 was described to co-sediment with the 40S ribosomal subunit in actively translating ribosomes, as polysomes, in normal and cancer cells, opening a new view on its involvement in tumor progression [17]. Lastly, the role of SERBP1 RNA-binding protein in pathways associated to cancer development was reported in glioblastoma [18].

Here, we discovered that Serbp1 is an Ago2-associated protein in the SHSY5Y neuronal-derived cell line. We also found a relationship between SERBP1 and Ago2 in the post-transcriptional regulation of the K^+^ Cl^−^ cotransporter KCC2, in human SH-SY5Y cells and in rat primary hippocampal neurons. KCC2 functions in setting the proper level of intracellular Cl^−^ ions by transporting Cl^−^ against a concentration gradient [19]. KCC2 is a fundamental regulator of GABA-mediated hyperpolarization, and it is involved in cortical development and plasticity [20]. KCC2 is also a multifunctional protein able to regulate actin cytoskeletal dynamics during development and synaptic plasticity [21]. KCC2 functionality may be modulated by post-translational modifications and also at the transcriptional and posttranscriptional levels [21]. Here we show that SERBP1 as well as Ago2/miR-92 complex bind to KCC2 3′UTR, and that miR-92 post-transcriptional regulation of KCC2 3′UTR is modulated by SERBP1 in neuronal cells. Together, these results show a novel mechanism of post-transcriptional regulation of KCC2 in neuronal cells, mediated by RNA-binding protein SERBP1.

## 2. Materials and Methods 

### 2.1. Plasmid SPORT6-MycSERBP1

The PCMV-SPORT6 eukaryotic expression vector containing SERBP1 cDNA was purchased from Open Biosystem,(Walthman, MA, USA). (MHS1010-7429844; IMAGE:4477452, complete cds. https://www.ebi.ac.uk/BC026916, 18 January 2022, EMBL-EBI,). The sequence coding for the c-Myc epitope was cloned into the BstEII site at the 5′ end of the SERBP1 coding sequence. pCDNA3-MycSERBP1: The MycSERBP1, derived from SPORT6-MycSERBP1 (EcoRI/NotI), was cloned into pCDNA3 commercial vector (EcoRI/NotI). PSSG: control firefly luciferase no containing 3′UTR was derived from SG-Luc-SLC12A5. Serpine1 3′UTR: The last 1332 nts of the Serpine1 3′UTR within the full-length clone SC119781 (Origene, Rockville, MD, USA) (SacII/NotI) and the first 509 nts of the Serpine1 3′UTR within the SG-Luc- Serpine1 plasmid (XbaI/SacII) (Amplicon start: chr7: 100362990; Amplicon end: chr7: 100363963 purchased from Switch Gear Genomic (Carlsbad, CA, USA) were cloned into the PSSG plasmid. APP 3′UTR: APP 3′-UTR was purchased from Switch-Gear Genomics (Carlsbad, CA, USA) (SG-Luc-APP; Amplicon start: chr21:26174703; Amplicon end: chr21:26175879). PSSG-KCC2 3′UTR: The KCC2 3′UTR (SG-Luc-SLC12A5; Amplicon start: chr20: 44119626; Amplicon end: chr20: 44122225) was purchased from Switch Gear Genomic (Carlsbad, CA, USA). BtKS-3′UTRKCC2 and BtSK-3′UTRKCC2: The full-length KCC2 3′UTR, derived from PSSG-KCC2 3′UTR (XbaI/XhoI), was cloned into Blue Script KS or SK vectors (XbaI/XhoI). 

### 2.2. Cell Cultures

SH-SY5Y and FLAG-Ago2 stable SH-SY5Y [8] cells were grown in Dulbecco’s Modified Eagle’s Medium supplemented with 10% fetal bovine serum, 100 unit/mL penicillin and 100 µg/mL streptomycin at 37 °C in 5% CO_2_. Transfection of cells was performed with 20 nM of SERBP1 siRNA duplexes or control siRNAs conjugated to Lipofectamine 2000 (Thermo Fisher Scientific, Walthman, MA, USA). Primary hippocampal neurons were prepared from day 17–18 embryos obtained from timed-pregnant Wistar rats (Charles River, IT, Wilmington, MA, USA). Neurons were plated on tissue culture dishes pre-coated with poly-lysine and cultured in neurobasal medium supplemented with B-27 and L-Glutamine (Gibco Walthman, MA, USA).

### 2.3. Immunoprecipitation

Cells were plated in 150 mm plates until a confluence of 90%, washed with ice-cold PBS and incubated for 30 min at 4 °C with 1mL of lysis buffer (0.5% NP40; 25 mM Tris-HCl pH 7.4; 150 mM KCl; 2 mM EGTA; 0.5 mM DTT; 10% Glycerol; 1:100 Protease inhibitor cocktail). Cells were then centrifuged for 10 min (1000× *g*) at 4 °C. Beads anti-FLAG M2 agarose conjugated, (Sigma-Aldrich St.Louis, MO, USA Cat# A2220, RRID: AB_10063035), or anti-Myc agarose conjugate (Sigma-Aldrich St.Louis, MO, USA Cat# A7470, RRID: AB_10109522) were washed with PBS, equilibrated with 1mL of lysis buffer and centrifuged at 4 °C for 6 min (1000× *g*). The supernatant of cell lysate was added to the beads and incubated at 4 °C for 4 h. After centrifugation for 10 min (1000× *g*), the supernatant was discarded and the beads were washed 3 times with 1mL of washing buffer (20 mM Tris-HCl pH 8.0; 0.1 M KCl; 5 mM MgCl_2_; 0.1% Tween 20; 10 mM β-mercaptoethanol; 10% Glycerol; 1:100 Protease inhibitor cocktail). An amount of 150 µL of epitope elution buffer (washing buffer containing FLAG peptide or c-Myc peptide at a concentration of 200 µg/mL) was added to the beads followed by incubation at rt for 2 h. Samples were centrifuged at 1000× *g* for 10 min and supernatants containing fusion protein and its interactors were recovered.

### 2.4. Protein Identification by LC-MS/MS

Anti-FLAG immunoprecipitation (IP) samples were separated by SDS-polyacrylamide gel electrophoresis (SDS-PAGE) 12%. Gels were stained by Comassie and bands of interest were manually excised from the gel. The proteins were reduced and alkylated using solutions of 10 mM DTT and 55 mM iodoacetamide and digested with tripsin. An amount of 6 µL of the peptide-containing solutions were injected in a CapLC system (Micromass, Waters Corporation, Milford, MA, USA) coupled online with a nano-ESI-Q-TOF instrument (Micromass, Waters Corporation, Milford, MA, USA). Samples were first trapped in a Waters Nanoease Atlantis C18 5 µm Trap Column and subsequently eluted on a Nanoease Atlantis Column (Waters Corporation, Milford, MA, USA) (75 µm × 100 mm), with a water/ACN gradient in the presence of 0.1% of formic acid. Raw data files were automatically processed into peaklist files by using MassLynx server v4.0 to obtain a Pkl format compatible with the MASCOT MS/MS ion search engine (http://www.matrixscience.com, 18 January 2022) with the NCBInr database restricted to “*homo sapiens*”. The estimation of false positive rate was calculated according to the Mascot default threshold (5%) to validate protein identification obtained by Peptide Mass Fingerprinting and by LC–MS/MS. The query was performed with the maximal tolerance for parent masses of 50 ppm and a maximal tolerance for fragments of 0.2 Da, searching peptides on 2+ and 3+ charge states. At the most, one missed cleavage for tryptic peptides was allowed, and the modifications accepted were carboamidomethylation with iodoacetamide of cysteines and oxidation of methionines. 

### 2.5. Western Blot Analysis

Lysis buffer (1% Triton, 0.1% SDS, 0.5% deoxycholic acid, 50 mM NaPO_4_, 150 mM NaCl, 2 mM EDTA) supplemented with protease inhibitor mixture (Sigma-Aldrich St.Louis, MO, USA) was used to extract protein from cultured cells. The samples were separated by SDS polyacrylamide gel electrophoresis, transferred to nitrocellulose membranes (Hybond ECL, GE Healthcare, Chicago, IL, USA) and incubated with appropriate primary and secondary peroxidase-coupled anti-rat, anti-mouse or anti-rabbit antibodies. Immunoreactivity was detected by chemiluminescence visualization and analyzed by Quantity One (Bio-Rad, CA, USA) software. Data are representative of three or four independent experiments. The following antibodies were used: mouse monoclonal anti-Glyceraldehyde 3-phosphate dehydrogenase (GAPDH) (1:4000; Covance, Princeton, NJ, USA; Cat# MMS-580S-200, RRID: AB_291468), rat monoclonal anti-Ago2 (1:1500, Thermo Fisher Scientific, MA, USA, Cat# 14-6519-82, RRID:AB_2784637), mouse monoclonal anti-SERBP1 (1:1500; Abnova, GmbH, Cat# H00026135-M01, RRID:AB_425924), mouse monoclonal anti-FLAG (M2) (1:1000; Sigma-Aldrich Cat# F3165, RRID:AB_259529) and rabbit polyclonal anti-KCC2 (1:1000; Millipore, MA, USA, Cat# 07-432; RRID:AB_310611). Assessing the performance of antibodies in genetic knockdown protein samples validated the specificity of Ago2 and Serbp1 antibodies. 

### 2.6. RT-PCR Assay and RNA Isolation

Total RNA was isolated using Trizol (Invitrogen, Waltham, MA, USA) reagent according to the manufacture’s procedure. Total RNA, after DNase treatment was reverse-transcribed into cDNA using Superscript III Reverse Transcription (Invitrogen, MA, USA) and amplified with SensiMix SYBR Hi-ROX (Bioline, Waltham, MA, USA) in a 7900HT Standard Real-Time PCR System (Applied Biosystems, Waltham, MA, USA). Probes detecting TATA-binding protein (TBP), Ago2 and Serbp1 were chosen from the Roche Universal Probe Library (Roche, CH) and used as recommended by the manufacturer. TBP (hsa TBP FW 273 GAACATCATGGATCAGAACAACA; hsa TBP REW 359 ATAGGGATTCCGGGAGTCAT); Ago2 (hsa AGO2 FW 1009 CCACCTCCCATGTTTACAAGTC hsa AGO2 REW 1085 CTGCCACAATGTTACAGACCTC); SERBP1 (hsa SERBP1 FW GAGGACGAGGTGGACGTG; hsa SERBP1 REW GGAGCAGAAGCACTTGACTTG)**;** KCC2 3′UTR (hsa KCC2 FW 1406 CTTCCCCAGCTCATCCTTG; hsa KCC2 REW 1447 GACAGAGCACGCCTCAGAC). Relative changes in gene expression were quantified using the comparative threshold method (Ct) after determining the Ct values for reference (TBP) and target genes in each sample set according to the 2^−ΔΔCt^ method. All reactions were performed in triplicate. 

### 2.7. RT- Quantitative PCR for miR-92 

The Taqman microRNA reverse transcription kit (Applied Biosystems, Waltham, MA, USA) and the TaqMan Universal PCR Master Mix II, no AmpErase UNG (Applied Biosystems, Waltham, MA, USA) were used. The U6 snRNA was used for normalization of samples. The quantitative PCR procedure was carried out according to the instructions provided with the TaqMan microRNA assay kit (Applied Biosystems, Waltham, MA, USA).

### 2.8. Luciferase Assay 

SH-SY5Y cells were plated at a density of 1 × 10^5^ per well in 24-well plates and transfected after 24 h with 20 nM of siRNA duplexes conjugated to 0.5 mL of Lipofectamine 2000 (Invitrogen, Waltham, MA, USA). After 48 h from siRNA transfection, 20 ng (for PSSG-APP 3′UTR) or 30 ng (for PSSG-SERPINE 3′UTR and PSSG-KCC2 3′UTR) of firefly luciferase expression vector and 5 ng of Renilla luciferase expression vector (pRL-TK Promega, WI, USA) conjugated to 0.5 mL of Lipofectamine 2000 were transfected. After 24 h cells were lysed and luciferase assays were performed using the Dual Luciferase Reporter Assay System (Promega, WI, USA) according to the manufacturer’s protocol. The experiments were carried out in triplicate. Hippocampal neurons were plated at a density of 2 × 10^5^ per well in 24-well plates and transfected after 72 h with 200 nM of siRNA duplexes (Invitrogen) and after 72 h from siRNA transfection with 40 ng of firefly luciferase expression vector and 4 ng of Renilla luciferase expression vector (pRL-TK Promega, WI, USA) conjugated to 2 µL of Lipofectamine 2000 (Invitrogen, Waltham, MA, USA). After 24 h, cells were lysed and luciferase assays were performed. The experiments were carried out in triplicate.

### 2.9. Ribonucleoprotein Immunoprecipitation 

SH-SY5Y cells were plated at a density of 3 × 10^6^ per 90 mm dish and after 24 h were transfected with either pCDNA3 control vector or pCDNA3-MycSERBP1. Proteins and associated mRNAs were isolated through immunoprecipitation of Myc-SERBP1 by modification of previously published protocols [22].

#### 2.9.1. Preparation of mRNP Lysate

After 48 h from transfection, cells were collected by centrifugation (1000× *g*) at 4 °C for 10 min and washed several times with 10 mL of ice-cold PBS. Cell pellets were lysated with an equal volume of polysomal lysis buffer (100 mM KCl, 5 mM MgCl_2_, 10 mM HEPES pH 7.0, 0.5% NP-40) with protease inhibitor cocktail and freshly added 1 mM DTT, 100 U/mL RNase inhibitor (RNaseOut, Invitrogen, Waltham, MA, USA) and 400 mM Vanadyl ribonucleoside complexes (Sigma-Aldrich). mRNP lysate was incubated on ice for 5 min and stored at −80 °C. mRNP lysates were thawed on ice and centrifuged at 15,000× *g* for 15 min to clear lysate of large particles. Cleared supernatants were transferred to new tubes and stored on ice. 

#### 2.9.2. Preparation of Beads

Anti-C-Myc-agarose conjugated beads (Sigma-Aldrich) were pre-incubated in NT2 buffer (50 mM Tris-HCl pH 7.4, 150 mM NaCl, 1 mM MgCl_2_, 0.05% NP-40) supplemented with 5% BSA for at least 1 h before use. After several washes, beads were resuspended in 850 μL of ice-cold NT2 buffer with 200 units of RNase inhibitor (RNase Out, Invitrogen, Waltham, MA, USA), 400 mM of Vanadyl ribonucleoside complexes (Sigma-Aldrich), 10 μL of 100 mM DTT and EDTA to 20mM.

#### 2.9.3. Immunoprecipitation Reaction and RNA Precipitation

For immunoprecipitation (IP), equal amounts of proteins (2–5 mg) corresponding to 100 μL of cleared lysate were incubated with 850 μL of NT2/Anti-C-Myc-agarose conjugated beads and tumbled for 4 h at 4 °C, followed by washing (4–5 times). The beads were resuspended in 100 μL of NT2 buffer supplemented with proteinase K and incubated at 55 °C for 30 min. RNA was recovered from Myc-IP by adding Tri-Reagent LS directly to the beads. Precipitation of RNA was carried out with the addition of glycogen (20 μg) to make the RNA pellets more readily visible. Recovered RNA was resuspended in nuclease-free water, measured for RNA concentration, and immediately reverse-transcribed into cDNA using random primers. RT-qPCR was carried out as described above. To examine proteins immunoprecipitated, the washed IP beads were incubated in sample buffer at 95 °C for 5 min and subjected to SDS-PAGE electrophoresis and immunoblotting.

### 2.10. RiboTrap Kit 

Ribonucleoprotein immunoprecipitation was also performed with a Ribo Trap Kit according to the manufacturer’s procedure. Briefly, BrU-labeled KCC2 sense and antisense 3′UTR transcripts were synthetized using T7 RNA polymerase (TranscriptAid T7 High yeld Transcription kit, ThermoFisher Scientific, Waltham, MA, USA) and plasmids BtKS-3′UTRKCC2 and BtSK-3′UTRKCC2, respectively. An amount of 50 pmol of BrU-labeled RNA was bound to anti-BrDUTP antibody conjugated to protein G agarose beads. Cytoplasmic lysates of SHSY cells were incubated with BrU-labeled RNA on antibody-conjugated beads for 2 h at 4 °C. After several washes with a mild buffer, the beads were incubated with 5-Bromo-2′-deoxyuridine to elute BrU-RNA/protein complexes. The proteins present in the complexes were analyzed by Western blotting. RNA present in the eluates was purified using Tri Reagent LS. Precipitation of RNA was carried out with the addition of glycogen (20 μg) to make the RNA pellets more readily visible. RT-qPCR for miR-92a was carried out as described above.

### 2.11. Statistics

Statistical analyses were performed using GraphPad Prism version 7 (GraphPad Software, San Diego, CA, USA): normality was tested by the Shapiro–Wilk normality test. Student’s *t*-test was used to evaluate significance of results. Statistical differences were considered significant with *p* < 0.05.

## 3. Results

### 3.1. Ago2 Interacts with SERBP1 in SH-SY5Y Neuroblastoma Cells

**Figure 1:**
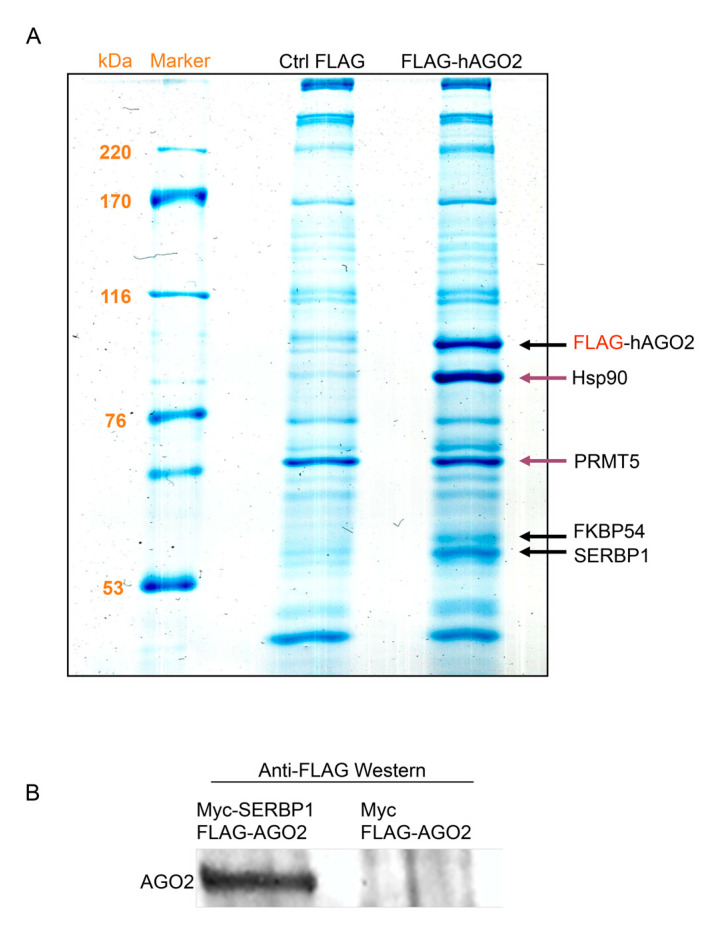
Ago2 interacts with SERBP1. (**A**) SDS-PAGE stained with Comassie Brilliant Blue of IP with FLAG antibody in SH-SY5Y stably expressing FLAG (lane 1) and FLAG-Ago2 (lane2). Bands corresponding to Ago2-associated proteins are indicated. (**B**) Cells were co-transfected with Myc-SERBP1 and Flag-Ago2 (lane 1) or with Myc and Flag-Ago2 as the negative control (lane 2) and immunoprecipitation was performed with anti-Myc antibody conjugated beads. Flag-Ago2 was detected by Western blot using anti-FLAG monoclonal antibody.

Ago2 protein is a fundamental component of effector complexes (RISC/miRNP complex) of silencing mechanisms. As an increasing amount of evidence in different organisms indicates that Argonaute proteins are involved in brain development and neuronal function, we tried to determine the composition of protein complexes containing hAGO2 in a neuronal-derived cell line. We used SH-SY5Y cells stably transfected with FLAG vector (Ctrl FLAG) or FLAG-hAGO2 construct [8]. We performed coimmunoprecipitation experiments of FlagAgo2 by the FLAG affinity strategy. Anti-FLAG immunoprecipitation (IP) samples were separated by SDS-PAGE 12%. Significant bands of Comassie-stained gels, corresponding to FLAG-hAGO2 samples, were identified by LC-MS/MS analysis (Figure 1A and Table S1). 

Thus, we have identified hAgo2-interacting proteins in this cell line. We confirmed Hsp90 as a known partner of hAgo2 [23], therefore validating the overall procedure. SERBP1 and FKBP5 were also identified as interaction partners of hAGO2 in Flag-AGO2-SH-SY5Y cells. The Hsp90 co-chaperone Fkbp5 (also known as Fkbp51 or Fkbp54), regulates glucocorticoid receptor activity via a negative feedback loop and it is a risk factor for several psychiatric disorders [24]. Interestingly, Fkbp5 has also been identified as an Ago2-associated protein in mouse embryonic stem cells [25]. SERBP1 was previously isolated in Ago2 protein complexes in mammalian cells [26,27]. Moreover, we identified a likely adventitious protein PRMT5 that systematically binds both to the empty vector and to Flag-Ago2. However, PRMT5 was previously reported as an interaction partner of hAgo2 [28] and recent studies show that it regulates arginine methylation of Ago2 [29]. 

Among Ago2 co-eluted proteins separated by SDS-PAGE and analyzed by mass spectrometry, we focused our research on the RNA-binding protein SERBP1. To confirm protein–protein interaction specificity an IP assay, using SERBP1 to isolate Ago2, was performed. SERBP1 was tagged with an N-terminal c-Myc epitope. Both Myc-SERBP1 and FLAG-Ago2 plasmids were co-transfected in human neuroblastoma cells SH-SY5Y; as a control we performed a co-transfection using a plasmid that expressed only c-Myc tag and the plasmid containing FLAG-Ago2. Myc-SERBP1 was immunoprecipitated using anti-Myc beads and proteins were eluted and separated by SDS-PAGE. The presence of FLAG-Ago2 in IP samples was revealed by Western blot, using anti-FLAG antibody. FLAG-Ago2 was observed in IP samples from Myc- SERBP1 co-transfected cells (Figure 1B), but was absent in control IP samples (Figure 1B). These results confirm the specificity of interaction between Ago2 and SERBP1 in SH-SY5Y cells.

### 3.2. RNAi of SERBP1 Reduces Ago2 Protein 

**Figure 2:**
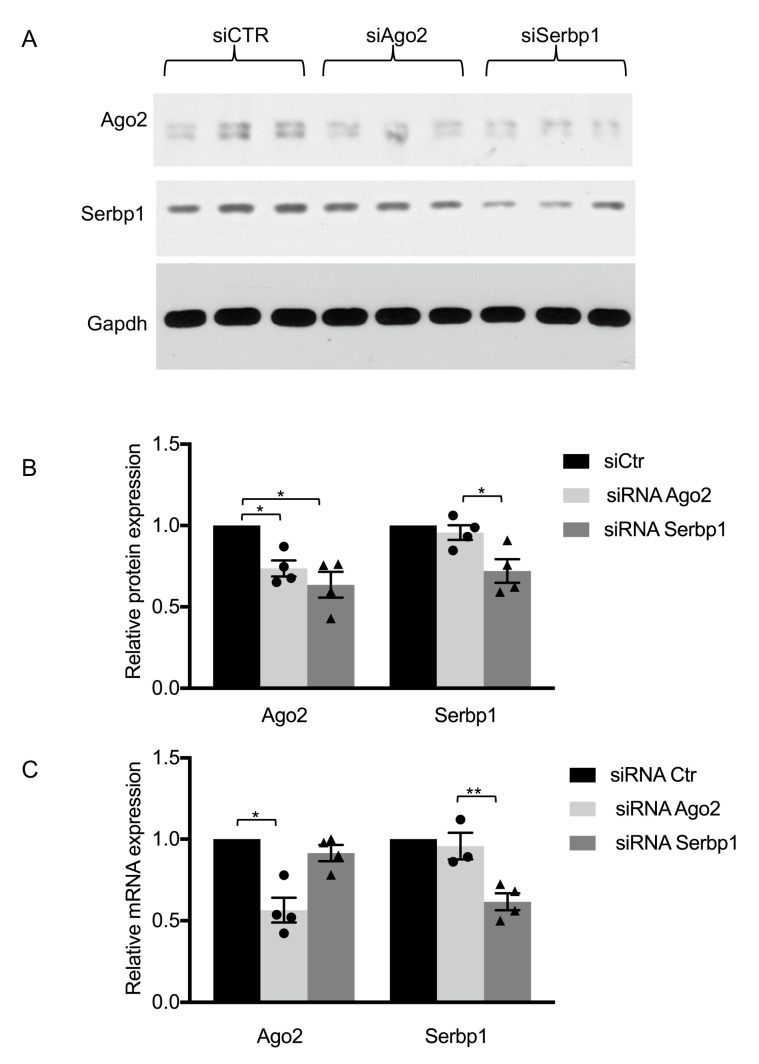
SERBP1-silencing downregulates Ago2 protein levels in SH-SY5Y cells. (**A**) Representative Western blot of extracts from cells transfected with control siRNA, Ago2 siRNA or SERBP1 siRNA from three independent experiments. Nitrocellulose membranes were hybridized with Ago2 or SERBP1 antibodies, as indicated. The GAPDH signal was used to normalize different samples (**B**). Fold change of Ago2 and SERBP1 protein levels in siRNA transfected cells relative to control siRNA samples are shown. Data represent the mean of three independent experiments ± standard error (S.E.); * *p* < 0.05 (*t*-test).. (**C**) RT-qPCR analysis of RNA extracted from SH-SY5Y cells transfected with control siRNA, Ago2 siRNA or SERBP1 siRNA. ΔCt values were normalized to TBP level. Fold change of Ago2 and Serbp1 mRNA expression levels relative to control siRNA samples are shown. Individual data points for Ago2 (circles) and Serbp1 (triangles) siRNA transfected cells are indicated. Data represent the mean of three independent experiments ± S.E.; * *p* < 0.05, ** *p* < 0.01.

It has been shown that Ago protein interactions [23,28,30,31] may affect Ago2 protein levels. To study whether SERBP1 may regulate Ago2 protein expression, RNAi-mediated depletion of SERBP1 was performed in SH-SY5Y cells. Cells were transfected with either a control or SERBP1 siRNA for 72 h. RNAi-mediated knockdown of Ago2 was used as a positive control. Interestingly, Western blotting revealed that Ago2 protein levels were reduced to 0.6 of control levels in samples where SERBP1 was silenced (Figure 2A,B). RT-qPCR of total RNA collected from SH-SY5Y cells showed that Ago2 mRNA levels were unchanged after RNAi of SERBP1 (Figure 2C). 

The reduction in Ago2 protein levels upon SERBP1 silencing was further validated in FLAG-Ago2-SH-SY5Y cells. Western blot with Flag antibody also showed that Flag-Ago2 levels were reduced upon SERBP1 silencing in FLAG-Ago2-SH-SY5Y cells (Figure S1). 

Taken together, these results indicate that SERBP1 silencing regulates Ago2 expression in neuronal cells and prompted us to investigate potential RNA molecules targeted by SERBP1/Ago2 complex.

### 3.3. SERBP1 Regulates KCC2 Expression through the 3′UTR in Neurons

**Figure 3:**
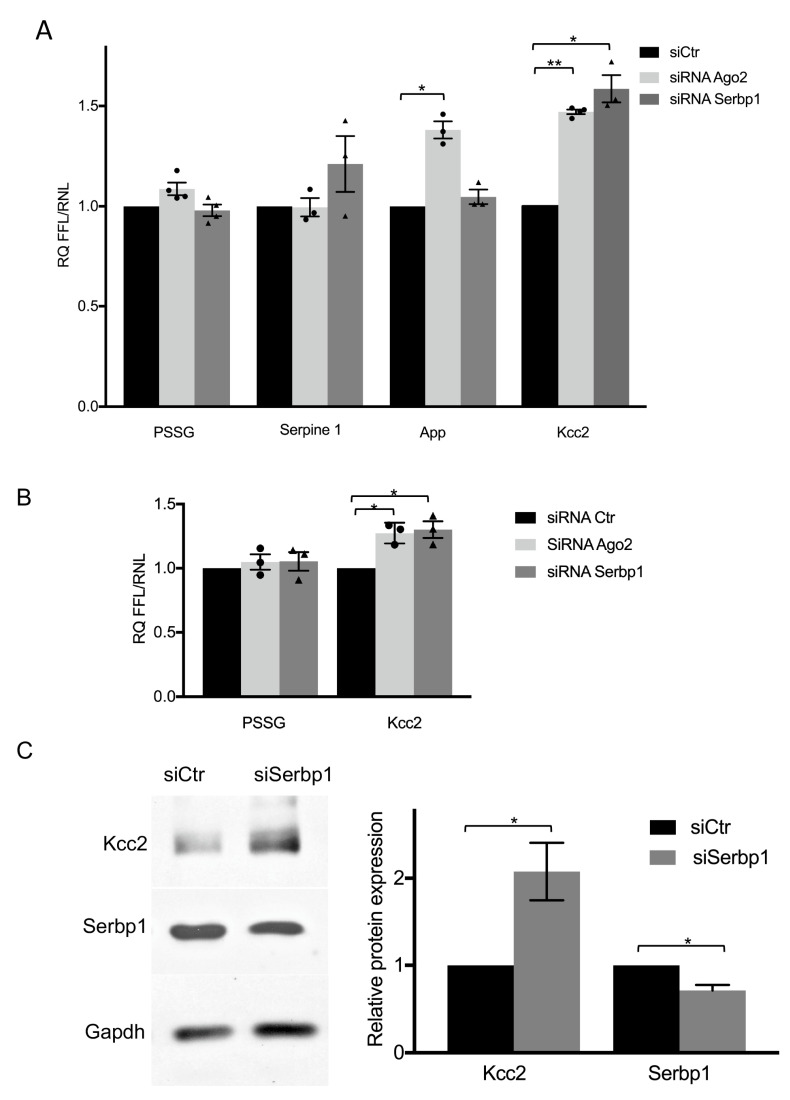
SERBP1 or Ago2 silencing increases KCC2 expression. (**A**) Forty-eight hours after transfection with either control siRNA, Ago2 siRNA or SERBP1 siRNA (20 nM), SH-SY5Y cells were transfected with PSSG control plasmid or the Serpine1, or APP or KCC2 3′UTR firefly reporter plasmid. Firefly/renilla luminescence ratios were determined 24 h later. The data are presented as the fold increase of normalized luminescence ratios relative to cells transfected with control siRNA. Data represent the mean of three independent experiments +/− S.E.; * *p* < 0.05; ** *p* < 0.01. (**B**) Forty-eight hours after transfection with siAgo2 or siSERBP1 or a control siRNA (200 nM), hippocampal neurons were transfected with control plasmid or KCC2 3′UTR luciferase reporter. Firefly/renilla luminescence ratio was analyzed as described in (**A**). Data represent the mean of three independent experiments ± S.E. * *p* < 0.05. Individual data points for Ago2 (circles) and Serbp1 (triangles) siRNA transfected cells are indicated in (**A**) and (**B**) panels. (**C**) Representative Western blotting showing KCC2 upregulation in hippocampal neurons after SERBP1 silencing. Nitrocellulose membranes were hybridized with KCC2 or SERBP1 antibodies, as indicated. The GAPDH signal was used to normalize different samples. Means +/− S.E. of KCC2 and SERBP1 protein levels obtained from three independent experiments are presented relative to control cells. * *p* < 0.05; ** *p* < 0.01 (*t*-test).

As SERBP1 was described as an RNA binding protein [9,32] and here we shown its interaction with Ago2, we wondered if SERBP1 is implicated in miRNA-mediated gene silencing of known target mRNAs in neuronal cells. Thus, we studied the effect of Ago2 or SERBP1 silencing on the expression of few firefly reporter vectors containing downstream of the firefly open reading frame the 3′UTR of selected miRNA targets in SH-SY5Y cells. We chose three representative molecules, with the intent to reveal a putative specific involvement of SERBP1/Ago2 complex in mRNA regulation. An obvious target, expected to respond to the silencing of SERBP1 or Ago2, was the Serpine1 3′UTR. A cyclic nucleotide-responsive sequence within the 3′UTR of the rat Serpine1 mRNA was indeed previously described to bind SERBP1 [9]. In addition, human Serpine1 3′UTR was found to contain several regulatory elements [33,34] and to be sensitive to miRNA-mediated post-transcriptional regulation [35]. Unexpectedly, we observed no changes in luciferase activity of the Serpine1 reporter after Ago2 silencing and a small but not statistically significant increase (20%) of luciferase activity after SERBP1 silencing (Figure 3A), suggesting that the RNA-binding protein may also act without RISC in this cellular context. The second tested luciferase reporter contained the APP 3′UTR, and it was analyzed because SERBP1 was described to be associated, in an RNA-independent manner, to a 52-base element downstream of the stop codon of APP 3′UTR [36]. Furthermore, APP 3′UTR was previously validated as a miRNA target (reviewed in [37]). We observed that the luciferase activity of APP 3′UTR reporter was increased (30%) by Ago2 silencing, as expected, whereas it was unaffected by SERBP1 depletion (Figure 3A). In this case, it is conceivable that the concomitant presence of SERBP1/Ago2 complex to repress APP mRNA is not necessary, but it is possible separately by alternative routes. Lastly, we analyzed an miRNA target, the KCC2 3′UTR [38], which until now was not described to interact with SERBP1. By luciferase assay, we found an increase (47% and 58%, respectively) of luciferase activity of KCC2 3′UTR reporter after silencing of either Ago2 or SERBP1 (Figure 3A). Our results suggest that SERBP1 functionally interacts with the KCC2 3′UTR to repress its expression. To verify this regulation in a physiological cellular context, as KCC2 is not expressed in SH-SY5Y cells but specifically expressed in neurons [39], we validated these results in cultured rat hippocampal neurons, a neuronal population widely used to study KCC2 biological functions. By luciferase assay we confirmed that silencing of either Ago2 or SERBP1 increased (27% and 31% respectively) the luciferase activity of KCC2 3′UTR reporter (Figure 3B). In neuronal extracts, we next observed that endogenous KCC2 protein expression was significantly upregulated after SERBP1 silencing in hippocampal neuronal cultures (Figure 3C). Our results show that SERBP1 expression levels may modulate KCC2 expression in neuronal cells, and suggest to investigate whether SERBP1 may participate in miRISC-mediated regulation of KCC2.

### 3.4. SERBP1, Ago2 and miR-92 Are Associated to the KCC2 3′UTR

**Figure 4:**
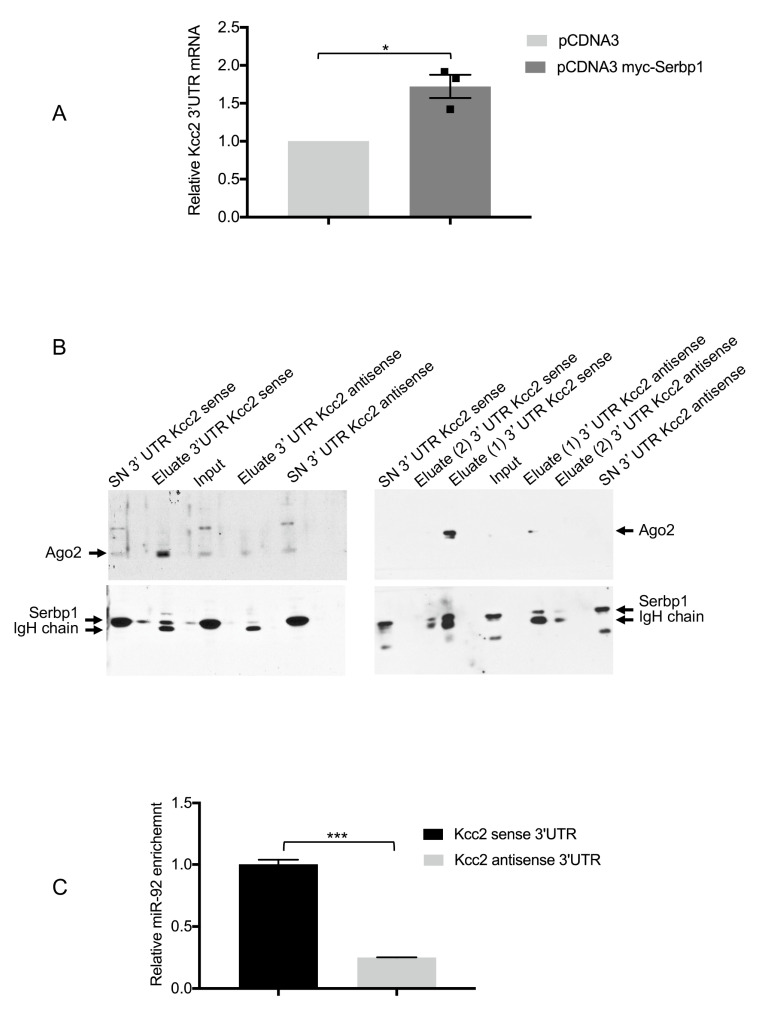
SERBP1 interacts with KCC2 3′UTR. (**A**) SH-SY5Y cells were co-transfected with pCDNA3-myc or pCDNA3 myc-SERBP1 together with KCC2 3′UTR. Immunoprecipitates were analyzed by qRT-PCR to determine the levels of KCC2 mRNA in the two different conditions. Data represent the mean of three independent experiments ± S.E.; * *p* < 0.05. Individual data points for pcDNA3 myc-Serbp1 (squares) transfected cells are shown. (**B**) Western blotting showing Ago2 and SERBP1 protein levels in the input, eluates and supernatants from cytoplasmic lysates of SH-SY5Y cells (left panels) or hippocampal neurons (right panels) incubated with KCC2 sense or antisense 3′UTR transcripts. Eluate (1) and eluate (2) indicate the first and second elution. (**C**) Levels of miR-92 bound to KCC2 sense or antisense 3′UTR transcripts were analyzed by qRT-PCR. ΔCt values were normalized to the snU6 level *** *p* < 0.001.

With the aim to demonstrate that SERBP1 may interact with the KCC2 3′UTR, a Ribonucleoprotein Immunoprecipitation and RT-qPCR assay were performed. Therefore, SH-SY5Y cells were co-transfected either with Myc-SERBP1 together with the KCC2 3′UTR reporter or pCDNA3 together with KCC2 3′UTR. We found that the KCC2 3′UTR was significantly enriched in SERBP1 immunoprecipitates compared to the control (Figure 4A). The SERBP1/KCC2 3′ UTR association was further validated by an RNA-protein binding assay. First, BrU-labeled KCC2 sense and antisense in vitro 3′UTR transcripts, bound to Anti-BrdU-agarose conjugated beads, were incubated with cytoplasmic lysates from SH-SY5Y cells or primary hippocampal neurons. Proteins associated to the 3′UTRs were eluted, fractionated by SDS-PAGE and analyzed by Western blot. Analysis of the specific signal for Ago2 and SERBP1 showed an enrichment of these proteins with the KCC2 sense 3′UTR (Figure 4B). These results suggest that both Ago2 and SERBP1 are specifically associated with the KCC2 3′UTR. At this stage we cannot establish whether SERBP1 is recruited on the KCC2 3′UTR by the interaction with Ago2 or instead it is directly bound to the mRNA. As we previously demonstrated that miR-92 may functionally interact with the KCC2 3′UTR [38], using the same RNA-protein binding assay described above and by RT-qPCR, we also analyzed miR-92 levels in the eluted complexes containing either the KCC2 sense or antisense 3′UTR. We observed an enrichment of miR-92 with the KCC2 sense 3′UTR (Figure 4C). These results suggest that SERBP1 is associated with the KCC2 3′UTR when an miRISC complex is assembled.

### 3.5. miR-92 Post-Transcriptional Regulation of KCC2 3′UTR Requires SERBP1

**Figure 5:**
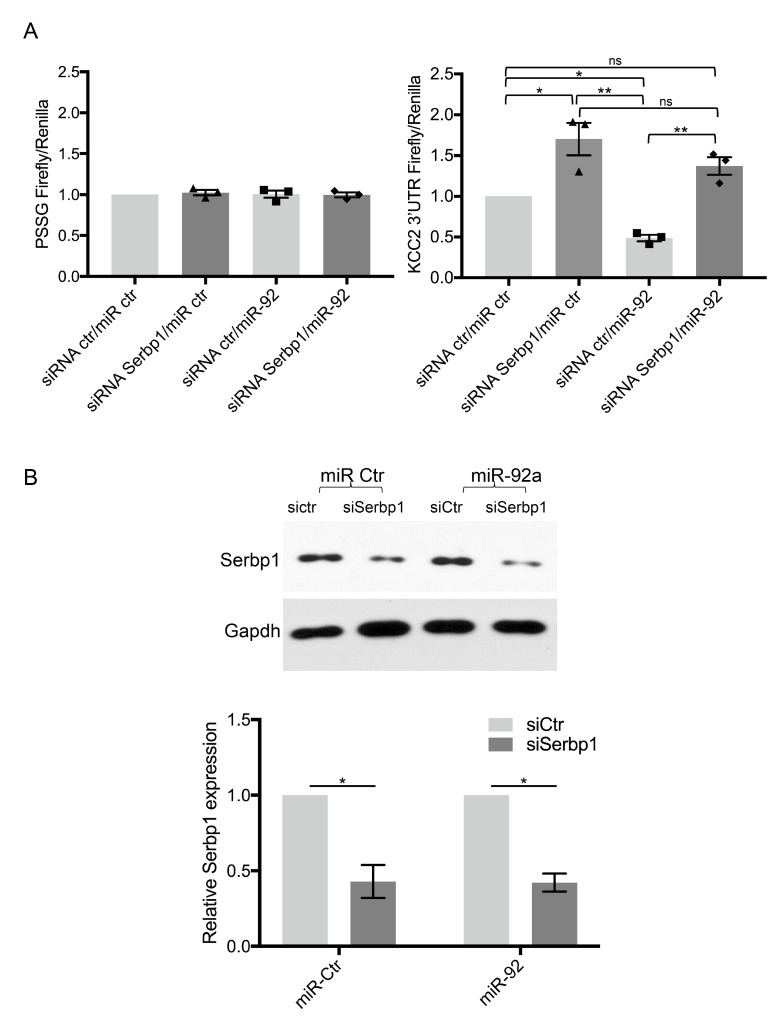
SERBP1 silencing relieves miR-92a-mediated translational repression of KCC2 3′UTR reporter. (**A**) Forty-eight hours after transfection with control siRNA or SERBP1 siRNA (20 nM), SH-SY5Y cells were co-transfected with PSSG control plasmid or the reporter with the 3′UTR of KCC2 together with a control miRNA or miR-92a. Firefly/renilla luminescence ratios were determined 24 h later. The data are normalized to cells transfected with control siRNA and control miRNA. Data represent the mean of three independent experiments ± standard error. ns: not significant, * *p* < 0.05; ** *p* < 0.01 (*t*-test). Individual data points for siRNA SERBP1/miR-Ctr (triangles), siRNA Ctr/miR-92 (squares), siRNA Serbp1/miR-92 (rhombus) co-transfected cells are shown. (**B**) Representative Western blot showing the efficiency of SERBP1 silencing in SH-SY5Y used for luciferase assay. Means +/− S.E. of SERBP1 protein levels obtained from three independent experiments are presented relative to control cells. * *p* < 0.05.

Effective repression of several miRNA targets may require the presence of Ago2 binding proteins [40]. Moreover, RNA binding proteins [41,42] may enhance the action of miRNAs/RISC on their targets [43]. To evaluate whether SERBP1 may influence miR-92/Ago2 activity, we analyzed whether the repressive effect of miR-92 on the expression of the KCC2 3′UTR reporter is affected upon depletion of SERBP1 in SH-SY5Y cells. We found that, when SERBP1 expression was reduced by 60%, miR-92 mediated suppression of KCC2 reporter was relieved (Figure 5). These results suggest that SERBP1 may assist the miR-92/Ago2 complex in the suppression of the KCC2 3′UTR target expression.

## 4. Discussion

In this study we investigated the interplay of SERBP1 and Ago2 in neuronal cells.

SERBP1 and Ago2 binding proteins, including Hsp90 and Fkbp54 that we identified by Ago2 IP/mass spectrometry in neuronal cells, have been previously listed in Ago2 interactome data sets [26,27]. Ago2 interacts with a variety of binding partners and appears to exist in multiple protein complexes. SERBP1 has been identified both in Ago2-Dicer and Ago2-Tnrc6c (a Dicer-independent) complexes [26,27].

Interestingly, SERBP1 silencing in neuronal cells leads to a reduction in Ago2 protein at the post-transcriptional level. However, the reduction in Ago2 levels, upon SERBP1 silencing, was not associated to a generalized reduction of miRISC activity. Indeed, we studied the effect of either Ago2 or SERBP1 silencing on the expression of 3′UTR firefly reporters of different mRNAs and we observed that although both App and KCC2 reporter were sensitive to Ago2 silencing, only the KCC2 reporter was modulated in both experimental conditions.

The lack of modulation of App upon SERBP1 silencing was puzzling. Indeed, previous studies have shown that SERBP1 may interact with the 3′UTR of App mRNA [36] and here we have shown that SERBP1 silencing reduces Ago2 levels. Therefore, we were expecting an effect on the App 3′UTR reporter. We can hypothesize that only unloaded Ago2 is reduced by SERBP1 silencing in our cellular model and therefore miRISC interaction with the App 3′UTR was unaffected. The function of SERBP1 in the RISC might be similar to Hsp90, which interacts and stabilizes Argonaute proteins without affecting miRNA-mediated regulation of targets [31]. Otherwise, SERBP1 or other RNA-binding proteins, which might compete with SERBP1, act as positive modulators of App translation balancing miRISC activity on the App 3′UTR. RNA binding proteins inhibiting miRISC access to mRNA targets through binding to or adjacent to the miRNA binding site have been reported [44,45]. In such cases, reduction of Ago2 due to SERBP1 silencing could be associated with a higher accessibility of the miRISC complex on the APP 3′UTR.

Regarding the novel finding that SERBP1 modulates Ago2/miR92-mediated KCC2 regulation in neuronal cells, we cannot establish whether SERBP1 is recruited directly on the KCC2 3′UTR or through Ago2. So far, the studies on KCC2 post-transcriptional regulation have focused on specific miRNAs targeting the 3′UTR. Indeed, we demonstrated that neuronal-expressed miR-92 is an endogenous fine regulator of contextual fear memory in mice [46]. We reported that miR-92-mediated post-transcriptional regulation in the hippocampus modulates contextual fear conditioning (CFC) memory, and memory-driven structural alterations in hippocampal neurons. CFC induces a transient hippocampal up-regulation of miR-92a, which leads to the downregulation of three miR-92 target genes, Cpeb3, Mef2D and KCC2. [46]. In addition, recently KCC2 was found to be a direct downstream target of miR-137 [47]. In an miR-137 conditional knock-out mouse, a neuropsychiatric model of anxiety-like behavior, neuronal KCC2 was significantly upregulated, suggesting that the miR-137-KCC2 pathway might be a potential target in neurological diseases associated with the deficiency of miR-137 [47]. Here we have demonstrated a novel layer of miRNA-mediated post-transcriptional regulation of KCC2 in neuronal cells, including the Ago2 interacting protein SERBP1. Further studies are required to investigate which other microRNAs/mRNAs targets can be regulated by SERBP1.

RNA-binding proteins play several roles in neuronal cells and the brain in physiological and pathological conditions [48]. Serbp1 expression levels modulate the transcription of genes linked to neurogenesis and synaptogenesis [18]. In addition, SERBP1 may interact with other RNA-binding proteins expressed in neuronal cells, such as FMR1, FXR1, FXR2 and SYNCRIP [49,50]. Thus, several Serbp1/protein complexes might be assembled and eventually impact the loading of miRNAs in RISC complexes or their subcellular localization. Notably, SYNCRIP has been shown to drive neural development by stabilizing specific pro-neural genes by direct interaction with mRNAs and repressing the translation of anti-neuronal mRNAs by miRISC complexes [51]. Thus, the multiplicity of these interactions and their cellular context could determine the fate of specific RNAs and the regulation of biological processes.

Regulation of KCC2 is of particular importance due to the critical role of this protein in neurological disorders including epilepsy, autism and Rett syndrome [52]. Remarkably in a Rett syndrome mouse model, at pre-symptomatic stages of disease, reduced expression of KCC2 and altered chloride homeostasis have been observed [53]. Furthermore, KCC2 RNA and protein expression levels are reduced in the post-mortem brain tissue of Rett syndrome patients [54]. An increase in SERBP1 protein levels has been reported in a model of Fragile X mental retardation [55], although the functional consequences have not yet been investigated. It might be interesting to explore the role of SERBP1/Ago2 complex and miR-92 on KCC2 regulation in Rett syndrome and other neurological disorders.

## Supplementary Materials

The following supporting information can be downloaded at https://www.mdpi.com/article/10.3390/cells11061052/s1, Table S1: List of proteins and corresponding peptides identified by LC-MS/MS analysis; Figure S1: Analysis of SERBP1 silencing in Flag-Ago2-SH-SY5Y cells.
