# Supplementary material for: Silencing of Ago-2 Interacting Protein SERBP1 Relieves KCC2 Repression by miR-92 in Neurons
Source: Cells. 2022 Mar 20;11(6):1052. doi: 10.3390/cells11061052 (PMC8947033; doi:10.3390/cells11061052)
Supplement: Supplementary file 1 [file cells-11-01052-s001.zip › cells-1624009-supplementary.pdf]

cells MDPI

Supplementary Materials

SERBP1 silencing relieves KCC2 repression by miR-92 in neurons

Barbato et al.

* Correspondence: christian.barbato@cnr.it (C.B.); carlo.cogoni@uniroma1.it (C.C.); francesca.ruberti@cnr.it (F.R.)

Table S1. List of proteins and corresponding peptides identified by LC-MS/MS analysis (see materials section for description).

NCBI accession number	Identified protein	Mascot score (for protein identification)	Identified peptides (Mascot ions score)
**gi|29171734**	**Ago2**<br>Nominal mass (M_r): 98400 Da; Calculated pI value: 9.32	**1644**	**LQANFFEMDIPK (79)**<br>**IDIYHYELDIKPEK (120)**<br>**TQIFGDR (34)**<br>**KPVFDGR (44)**<br>**NLYTAMPLPIGR (37)**<br>**LPSVPFETIQALDVVMR (114)**<br>**SFFTASEGCSNPLGGGR (114)**<br>**AQPVIEFVCEVLDFK (89)**<br>**HTYLPLEVCNIVAGQR (72)**<br>**ASFNTDPYVR (50)**<br>**VLQPPSILYGGR (56)**<br>**AIATPVQGVWDMR (78)**<br>**YAQGADSVEPMFR(67)**<br>**NTYAGLQLVVVILPGK (102)**<br>**VGDTVLGMATQCVQMK(98)**<br>**TTPQTLSNLCLK (64)**<br>**QEIIQDLAAMVR (81)**<br>**ELLIQFYK(41)**<br>**DYQPGITFIVVQK(54)**<br>**SGNIPAGTTVDTK (67)**<br>**SVSIPAPAYYAHLVAF(77)**
**gi|20149594**	**heat shock 90kDa**<br>Nominal mass (M_r): **83554**; Calculated pI value: **4.97**	657	**YESLTDPSK (61)**<br>**IDIIPNPQER (72)**<br>**TLTLVDTGIGMTK (67)**<br>**ADLINNLGTIAK (90)**<br>**ADHGEPIGR (40)**<br>**YIDQEELNK (61)**<br>**SLTNDWEDHLAVK (56)**<br>**ALLFIPR (45)**<br>**APFDLFENK (46)**<br>**LGIHEDSTNR (50)**<br>**FENLCK (42)**
**gi|20070220**	**protein arginine**	602	**DLNCVPEIADTLGAVAK (66)**

	**methyltransfer-ase (PRMT5)** Nominal mass (M_r): **73322**; Calculated pI value: **5.88**		**EFIQEPAK (44)** **DWNTLIVGK (46)** **VPLVAPEDLR (61)** **TLCDYSK (33)** **AAILPTSIFLTNK (95)** **KGFPVLSK (46)** **GPLVNASLR (37)** **DPMIDNNR (47)** **EGQTICVR (38)**
**gi|1145816**	**FKBP54** Nominal mass (M_r): **50859**; Calculated pI value: **5.80**	**1360**	**NNEESPTATVAEQGEDITSKK (110)** **VYVHYK (27)** **FDSSHDRNEPFVFSLGK (103)** **NEPFVFSLGK (46)** **AWDIGVATMK (65)** **IPSNATLFFEIELLDFKGEDLFEDG-GIIR (30)** **KGEGYSNPNEGATVEIHLEGR (147)** **DVAFTVGEGEDHDIPIGIDK (100)** **MQREEQCILYLGPR (53)** **MQREEQCILYLGPR Oxidation (M) (49)** **EEQCILYLGPR (46)** **YGFGEAGKPK (62)** **FGIEPNAELIYEVTLK (98)** **YMQAVIQYGK (57)** **ALGLDSANEK (81)** **RGEAQLLMNEFESAK (108)** **VLEVNPQNK (61)** **LQISMCQK (42)** **FAEQDAKEEANK (50)**
**gi|12803339**	**SERPINE1 mRNA binding protein 1** Nominal mass (M_r): **44291**; Calculated pI value: **8.31**	**77**	**EAGGGGVGGPGAK (53)** **AKVEFNIR (24)** **SAAQAAAQTNSNAAGK (50)**

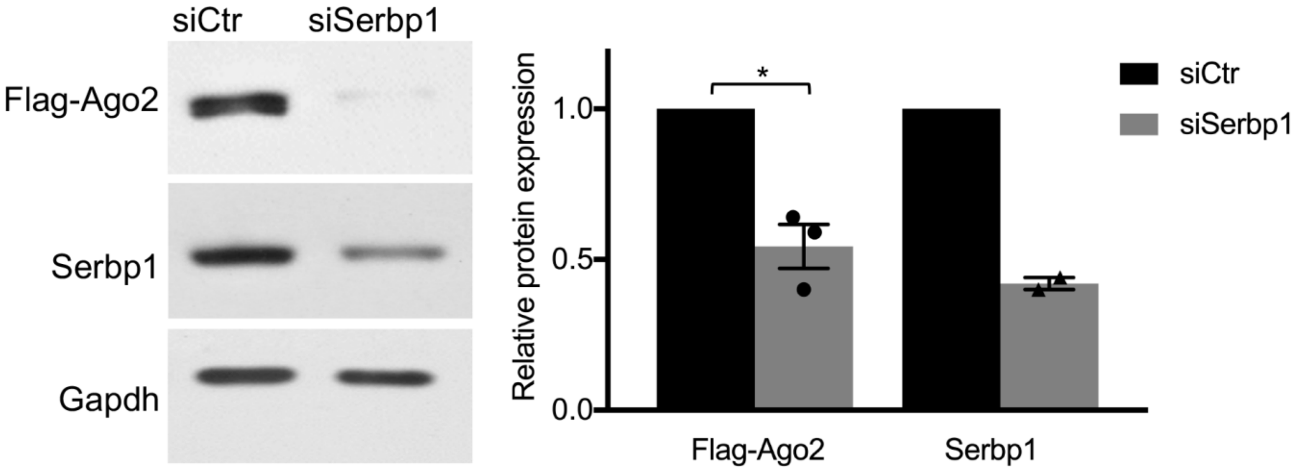

Figure S1. Analysis of SERBP1 silencing in Flag-Ago2-SH-SY5Y cells. Representative Western blotting showing Flag-AGO2 and SERBP1 protein levels. The GAPDH signal was used to normalize different samples. Fold change of proteins in SERBP1 silenced samples relative to control siRNA is shown. Individual data point for Flag-Ago2 (circles) and Serbp1 (triangles) proteins are indicated (Mean ± S.E; *—$p < 0.05$).
